# Intestinal Bacterial Colonization in the First 2 Weeks of Life of Nigerian Neonates Using Standard Culture Methods

**DOI:** 10.3389/fped.2016.00139

**Published:** 2016-12-27

**Authors:** Allan Kigbu, Adebola E. Orimadegun, Olukemi O. Tongo, Georgina N. Odaibo, David O. Olaleye, Olusegun O. Akinyinka

**Affiliations:** ^1^Department of Paediatrics, College of Medicine, University of Ibadan, Ibadan, Nigeria; ^2^Institute of Child Health, College of Medicine, University of Ibadan, Ibadan, Nigeria; ^3^Department of Virology, College of Medicine, University of Ibadan, Ibadan, Nigeria

**Keywords:** bacterial colonization, early life, newborns, preterm infants, rectal swabs

## Abstract

**Objective:**

The pattern and timing of development of intestinal microflora in Nigerian infants have been scarcely researched. This study was carried out to investigate the bacteria flora in the rectum of healthy neonates in Ibadan, Nigeria.

**Patients and methods:**

In this hospital-based longitudinal study, rectal swabs of 70 neonates were taken within 6–12 h of birth (day 1) and subsequently on days 3, 9, and 14. Information collected included maternal sociodemographic characteristics, antibiotic use for the neonates, and type of feeding during the first 14 days of life. Identification and speciation of gram-negative isolates were done using the Analytical Profile Index 20E^®^ and 20NE^®^ as appropriate. Gram-positive bacteria were identified biochemically using the catalase and coagulase tests. Data were analyzed using descriptive statistics and Chi-square at *p* = 0.05.

**Results:**

Majority (92.9%) of the neonates were delivered vaginally with a median gestational age of 38 weeks (range = 34–42). On the first day of life, *Escherichia coli* was isolated more frequently from the rectal swabs of preterm (50.0%) than term (23.1%) neonates (*p* = 0.031). On day 3 of life, coagulase-negative *staphylococcus* was the most frequently isolated bacteria from the rectal swabs of nonasphyxiated (64.4%) compared with asphyxiated (27.3%) neonates’ rectal swabs (*p* = 0.042). *Staphylococcus aureus* was the most frequently isolated bacteria from the rectal swabs of nonexclusively breastfed (66.7%) than exclusively breastfed (21.3%) neonates on day 14 (*p* = 0.004).

**Conclusion:**

*Staphylococcus aureus* and *Escherichia coli* were the predominant isolates from the rectum of Nigerian neonates, and these isolates were influenced by breastfeeding and mild–moderate asphyxia. In all, bacterial diversity in the rectum increased as the neonates got older.

## Introduction

The composition of intestinal bacterial flora is critical because of its role in nutrition, immune modulation, and biosynthesis of vitamins among other benefits (1). Currently, attention is on the health benefits and consequences of modification of bacterial composition of the intestine in early life. The introduction of pre- and probiotics, use of antibiotics, and experimental fecal flora transplantation to modify bacterial composition of intestine have improved health outcomes in necrotizing enterocolitis and antibiotic-associated diarrhea in children (2, 3). Studies have also associated diseases such as kwashiorkor, obesity, asthma, inflammatory bowel disease, and type 2 diabetes mellitus with pattern of bacterial microflora in early life (2, 4–6).

Consequently, the knowledge of the composition of the intestinal bacteria flora and its developmental pattern as well as possible alterations in different populations has become important in recent times. Notably, the development of intestinal bacterial population starts early in neonatal life and it is, to an extent, determined by the exposure of the baby to bacteria in the immediate environment (7, 8). Common environmental exposures include bacterial flora in maternal feces, vagina, and skin. Therefore, the types of bacterial flora acquired by a neonate are influenced by the mode of delivery, infant care practices such as type of feeding, the cleanness of the nursing environment, hospitalization, administration of antibiotics to the neonate, and maturity at the time of delivery (7, 9).

Currently, there are many techniques that can be used to identify bacterial composition of samples from the intestine including the traditional microbial culture, polymerase chain reaction, microarrays, and high-throughput sequencing (HTS). Of these, microbial culture is still considered the gold standard for clinical diagnostic purposes, and it is widely used in clinical laboratories (10). The HTS technologies that give better insights into the gut microbiota, because it can identify noncultivable organisms, are very specialized, expensive, and time consuming. These techniques are not available in resource-limited countries like Nigeria and they are not routinely used. However, a recent review by called attention to the fact that results of these technologies give correlate closely with those from culture-based approaches, showing the ability to corroborate and supplement the culture-based results (11).

Moreover, it is worth noting that to date, the successes recorded in the understanding of intestinal bacterial composition among infants are largely based on findings from studies conducted in developed countries (12), while data from developing countries like Nigeria remain scarce with available reports being over 30 years old (13, 14). With changes in lifestyle and the dynamic of factors that may influence intestinal colonization as well as development of better microbiological identification methods over time, it is probable that the intestinal microflora of Nigerian children might have changed. This study was, therefore, carried out to investigate the intestinal bacterial flora of neonates in Ibadan, Nigeria.

## Patients and Methods

### Study Design and Setting

This longitudinal study was carried out between August and December 2013. The neonates were recruited from the labor and postnatal wards of the University College Hospital (UCH), Ibadan within 6–12 h of birth (day 1). The neonates were subsequently seen on days 3, 9, and 14 after birth. The UCH provides primary and specialist care to Ibadan and other parts of the South-West region of Nigeria. The labor and postnatal wards have a total of 75 beds, where healthy neonates and those who do not require intensive care are nursed besides their mothers after delivery. In the absence of maternal and neonatal complications, neonates are usually discharged within 48–72 h after delivery.

### Study Population

All healthy neonates were considered eligible to participate in the study. A neonate was regarded as “healthy or needing no intensive care” if there were no clinical features indicating the need for admission into the special care baby unit, but well enough to be nursed by the mother’s bedside. Seventy-two neonates were enrolled at birth but two mothers declined further participation of their babies after the first day, giving a dropout rate of 2.8%.

### Sample Size Calculation

The required sample size was determined using the colonization rate from the previous study by Rotimi et al. (14) in 1985 which identified *Escherichia coli* as the commonest organism in 18 out of 23 babies by the first day of life, giving 78.2% colonization rate. It was hypothesized that the percentage of neonates with *Escherichia coli* colonization rate would be ±10% than the previously reported. At 95% level of confidence, 65 babies were required. Considering the fact that each neonate was sampled four times, 264 rectal swabs samples were expected.

### Sampling Technique

Systematic random sampling method was used to recruit every fifth live-born neonate in the UCH who met the inclusion criteria during the study period. Neonates who had anorectal anomalies were not eligible to participate in the study.

### Data Collection and Follow-Up

Two trained research assistants collected data using a structured interviewer-administered questionnaire. All rectal swab samples were collected by two of the investigators (AK and AEO). Information recorded on questionnaire included mothers’ demographic characteristics, pregnancy and delivery history; infant’s characteristics including APGAR scores, mode of resuscitation, feeding methods, and antibiotic/antimycotic use. The 5-min APGAR scores of ≤3, 4–6, and 7–10 were regarded as indicative of low (severe asphyxia), mild–moderate abnormal, and reassuring (no asphyxia), respectively (15). We determined the gestational ages of the neonates using the date of the last menstrual period (LMP) reported by mothers corroborated with ultrasound report. In the event the mother’s LMP was unknown, the gestational age of the baby was estimated by the pediatrician using the New Ballard score system (16). Preterm neonates were those whose gestational ages were less than 37 weeks.

Rectal swabs were taken from the neonates on the first day within 6–12 h after birth while on the ward. Subsequent samples were collected on days 3, 9, and 14 when mothers brought their babies for follow-up. Samples were collected in an aseptic manner using sterile gel coated soft cotton tipped swab sticks (Transwab^®^ Amies; Medical Wire & Equipment, Corsham, Wiltshire, England). The Transwab^®^ is a sterile pack containing a gel-coated soft cotton tipped swab and a container containing Amies transport medium and recommended for recovery of aerobes, anaerobes, and fastidious organisms. All specimens were collected following the procedures described in Centre for Diseases Control manual (17). Each tube containing the swab was placed in a transport pack and in a cold box and transported to the laboratory within 3 h after collection by a laboratory motorcycle dispatcher. Five mothers who failed to present their babies for follow-up at different times were located through their addresses and phone numbers with the rectal swabs samples collected through home visits.

### Microbiological Methods

The rectal swabs on arrival in the laboratory were plated onto MacConkey agar and MacConkey Crystal Violet agar alongside Salmonella Shigella agar plates. Each sample was also inoculated onto a Selenite F broth and further processed in accordance with the standard practice for bacteria isolation. Other media used were Columbia blood agar and Amikacin blood agar, which were both incubated anaerobically at 37°C for 2 days. Inoculation of the plates was carried out using standard methods such that a semiquantitative assessment of the density of growth could be made on the solid media. All primary plates were reincubated after 18 h, regardless of the culture results.

Upon isolation of organisms, gram staining was done, which divided isolates into gram-negative and -positive bacteria. The complete identification and speciation of all the gram-negative isolates were done using the Analytical Profile Index (API) 20E^®^ and API 20NE^®^ (API^®^ bioMérieux Clinical Diagnostics Kits, USA) identification system in accordance with the standard procedures for bacteria classification. The API^®^ is a miniaturized identification system that contains dehydrated sugars that gives a characteristic color change upon addition and incubation of substrates. Miniaturized reagents are also incorporated in the system. API 20E^®^ identified members of the *Enterobacteriaceae* while API 20NE^®^ identified the nonfermenters and biochemically inert gram-negative rods. Gram-positive cocci were identified biochemically using the catalase test and coagulase test; Pastorex for *Staphylococci* (18). Anaerobes were conclusively identified employing the use of AnaeroGen a miniaturized GasPak™ gas regenerating kit system (19). All plates were kept for 40 h before being read. Indicator strips were added into the anaerobic jars to ascertain the presence of anaerobiosis.

### Data Analysis

The main outcome was the frequency of the types of isolates cultured on days 1, 3, 9, and 14 while independent variables included characteristics of neonates and mothers. Chi-square test was used to test associations between categorical variables, while comparisons between continuous variables were performed using Student’s *t*-test. The patterns of organisms identified on the different days were displayed in charts. All *p* < 0.05 were considered statistically significant. The data were analyzed using Statistical Package for Social Sciences (SPSS) for Windows 21 (IBM SPSS statistics 2012, Chicago, IL, USA).

### Ethical Considerations

Approval for the study was obtained from the University of Ibadan/UCH Ethics Review Committee (approval number: UI/EC/13/0161), and written informed consent was obtained from each mother.

## Results

### Characteristics of the Mothers and Neonates

Of the 68 mothers whose neonates participated in the study, two had twin deliveries. The mothers’ age ranged from 17 to 40 years (mean = 28.5 ± 5.1 years). Twenty-six (38.2%) of the mothers were primipara, 65 (95.6%) booked and received antenatal care at the study center. Pregnancy-related medical conditions recorded in the hospital notes and corroborated by mothers’ reports were hypertension (*n* = 3; 4.4%), antepartum hemorrhage (*n* = 1; 1.5%), postpartum hemorrhage (*n* = 1; 1.5%), and polyhydramnios (*n* = 1; 1.5%). Only two (2.9%) mothers had PROMs both lasting >24 h and they were given intravenous Augmentin (amoxicillin and clavulanate) prophylactically for 48 h, then orally to complete a 7–10-day course. Three mothers (4.4%) had prolonged labor lasting 19 to 24 h. Sixty-six (97.1%) of the mothers reported no illness or complications during pregnancy, and only two (2.9%) were “HIV positive.”

Table 1 shows the characteristics of the neonates. There were 32 (45.7%) males and 38 (54.3%) female neonates. There were 66 singletons and two sets of twins. Sixty-one neonates (87.1%) were exclusively breastfed, and 9 (12.9%) were given water and/or formula in addition to breast milk. Two neonates required hospital admission, which occurred during the second week of life, with aspiration pneumonitis and acyanotic congenital cardiac defect, respectively. One mother reported administering oral antibiotics to her neonate (1.4%). Five (7.1%) neonates were delivered by elective cesarean section. One female term neonate had pustular skin rash on the third day of life was given a course of oral cephalexin, which resolved by the ninth day of life.

**Table 1:** **Baseline characteristics of neonates**.

	Male (*n* = 32)	Female (*n* = 38)
Number of neonates (%)	32 (45.7)	38 (54.3)
Number of singleton pregnancy	30 (45.6)	36 (54.4)
Duration of hospital stay (days)	2–4	2–3
Gestational age (weeks)		
Median (range)	37 (34–43)	38 (34–42)
Mean ± SD	37.0 ± 15.2	39.01 ± 10.12
Preterm, *n* (%)	8 (44.4)	10 (55.6)
Term, *n* (%)	24 (46.2)	28 (53.8)
Mode of delivery		
Vaginal	30 (46.2)	35 (53.8)
Cesarean section	2 (40.0)	3 (60.0)
Birth weight (g)		
Median (range)	3000 (1700–4100)	2825 (2000–3900)
Mean ± SD	3069.1 ± 560.3	2833 ± 409.4
LBW, *n* (%)	3 (60.0)	2 (40.0)
NBW, *n* (%)	29 (44.6)	36 (55.4)
APGAR score at 5 min		
Median (range)	7 (4–8)	7 (5–8)
Mild–moderate	7 (66.7)	4 (33.3)
No asphyxia	25 (41.7)	34 (58.3)

*LBW, low birth weight; NBW, normal birth weight*.

*Preterm, gestational age <37 weeks; Term, gestational age ≥37 weeks*.

*APGAR score at 5 min—a score of 7–10 was no asphyxia, a score of 4–6 was moderately abnormal, and a score of 0–3 was low*.

### Bacterial Isolates on the First Day of Life and Associated Factors

**Figure 1:**
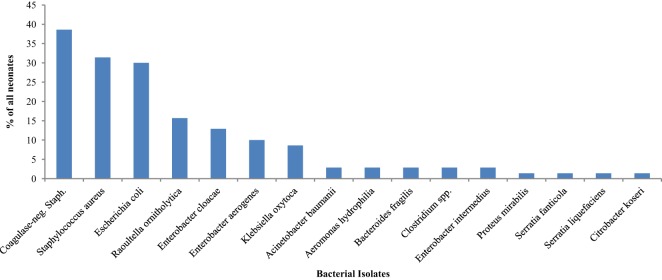
**Bacterial isolates from the rectum of neonates on the first day of life**.

Bacteria were isolated from the rectal swab samples of 65 (92.9%) on day 1 mainly facultative anaerobes (15 of 16 different species). Figure 1 shows the species of isolates. The most frequently isolated bacteria was coagulase-negative *Staphylococcus* (*n* = 27; 38.6%); followed by *Staphylococcus aureus* (*n* = 22; 31.4%) and *Escherichia coli* (*n* = 21; 30.0%). *Serratia fanticola, Serratia liquefaciens, Citrobacter koseri*, and *Proteus mirabilis* were found in samples from one neonate each. With the exception of *Escherichia coli* in preterm neonates (*n* = 9; 50.0%) compared with term neonates 12 (23.1) (*p* = 0.031), there was no significant differences in the bacterial isolates in preterm and term neonates (Table 1).

Table 2 also shows comparisons of frequency of bacterial isolates from the neonates between neonates who were preterm versus term, had mild to moderate asphyxia versus no asphyxia, and delivered per vaginal versus cesarean section. On the first day of life, the odds of isolating *Escherichia coli* (OR = 3.3; 95% CI: 1.1, 10.2) from the rectal swabs of preterm neonates was significantly higher than that of term neonates.

**Table 2:** **Frequency of 10 common bacterial isolates from the rectum of neonates on day 1 of life**.

Isolates	Maturity			Asphyxia			Mode of delivery		
	Preterm *N* = 18	Term *N* = 52	*p*	Yes *N* = 11	No *N* = 59	*p*	Vaginal *N* = 65	CS *N* = 5	*p*
*Acinetobacter baumannii*	1 (5.6)	1 (1.9)	0.451	0 (0.0)	2 (3.4)	0.536	2 (3.1)	0 (0.0)	1.000
*Aeromonas hydrophila*	1 (5.6)	1 (1.9)	0.451	0 (0.0)	2 (3.4)	0.536	2 (3.1)	0 (0.0)	1.000
*Bacteroides fragilis*	1 (5.6)	1 (1.9)	0.451	1 (9.1)	1 (1.7)	0.292	2 (3.1)	0 (0.0)	1.000
Coagulase-negative *Staph*	7 (38.9)	20 (38.5)	0.974	4 (36.4)	23 (39.0)	0.870	26 (40.0)	1 (20.0)	0.642
*Enterobacter aerogenes*	1 (5.6)	6 (11.5)	0.417	1 (9.1)	6 (10.2)	0.913	7 (10.8)	0 (0.0)	1.000
*Enterobacter cloacae*	1 (5.6)	8 (15.4)	0.265	2 (18.2)	7 (11.9)	0.566	8 (12.3)	1 (20.0)	0.508
*Escherichia coli*	9 (50.0)	12 (23.1)	0.031*	4 (36.4)	17 (28.8)	0.616	21 (32.3)	0 (0.0)	0.158
*Klebsiella oxytoca*	2 (11.1)	4 (7.7)	0.655	1 (9.1)	5 (8.5)	0.947	6 (9.2)	0 (0.0)	1.000
*Raoultella ornithinolytica*	1 (5.6)	10 (19.2)	0.160	1 (9.1)	10 (16.9)	0.837	10 (15.4)	1 (20.0)	0.586
*Staphylococcus aureus*	4 (22.2)	18 (34.6)	0.391	5 (45.5)	17 (28.8)	0.303	20 (30.8)	2 (40.0)	0.646

*Chi-square test was used to generate the p-values. Numbers in parenthesis are in %*.

*Odds ratio = 3.3 (95% CI: 1.1, 10.2).

### Bacterial Isolates the Third Day of Life and Associated Factors

**Figure 2:**
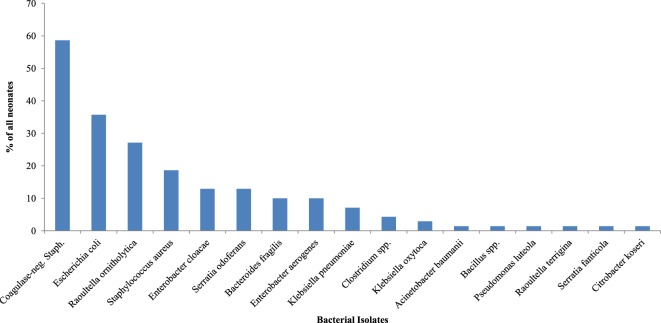
**Bacterial isolates from the rectum of neonates on the third day of life**.

Different species of isolates were obtained from all 70 (100.0%) rectal samples on day 3 of life (Figure 2). Coagulase-negative *Staphylococcus* (58.6%) was the most frequently isolated bacteria on day 3. Table 3 shows the bacteria isolates from neonates by maturity, presence or absence of asphyxia at birth, mode of delivery, and whether or not neonate was exclusively breastfed for the first 3 days of life. The coagulase-negative *Staphylococcus* was more frequently (*p* = 0.042) isolated in nonasphyxiated neonates (64.6%) than asphyxiated neonates (27.3%). Asphyxiated neonates had significantly higher colonization by *Enterobacter cloacae* (36.4%) than nonasphyxiated neonates (8.5%) (*p* = 0.029). On day 3, the odds of isolating coagulase-negative *Staphylococcus* from the rectal swabs of nonasphyxiated neonates were significantly higher than that of asphyxiated neonates (OR = 4.83; 95% CI: 1.15, 20.16). Asphyxiated neonates had significantly higher odds of colonization by *Enterobacter cloacae* (OR = 6.1; 95% CI: 1.33, 28.57) than nonasphyxiated neonates.

**Table 3:** **Frequency of 10 common bacterial isolates from the rectum of neonates on the third day of life**.

Isolates	Maturity			Asphyxia			Mode of delivery			Exclusive Breast feeding		
	Preterm *N* = 18	Term *N* = 52	*p*	Yes *N* = 11	No *N* = 59	*p*	Vaginal *N* = 65	CS *N* = 5	*p*	Yes *N* = 61	No *N* = 9	*p*
*Bacteroides fragilis*	2 (11.1)	5 (9.6)	0.855	2 (18.2)	5 (8.5)	0.302	7 (10.8)	0 (0.0)	1.000	6 (9.8)	1 (11.1)	0.905
*Clostridium* spp.	0 (0.0)	3 (5.8)	0.298	1 (9.1)	2 (3.4)	0.406	3 (4.6)	0 (0.0)	1.000	3 (4.9)	0 (0.0)	1.000
Coagulase-neg. *Staph*	10 (55.6)	31 (59.6)	0.487	3 (27.3)	38 (64.4)	0.042*	40 (61.5)	1 (20.0)	0.069	37 (60.7)	4 (44.4)	0.474
*Enterobacter cloacae*	2 (11.1)	7 (13.5)	0.797	4 (36.4)	5 (8.5)	0.029^+^	7 (10.8)	2 (40.0)	0.120	7 (11.5)	2 (22.2)	0.326
*Enterobacter aerogenes*	1 (5.6)	6 (11.5)	0.668	1 (9.1)	6 (10.2)	1.000	5 (7.7)	2 (40.0)	0.075	6 (9.8)	1 (11.1)	0.905
*Escherichia coli*	8 (44.4)	17 (32.7)	0.403	3 (27.3)	22 (37.3)	0.735	23 (35.4)	2 (40.0)	0.836	21 (34.4)	4 (44.4)	0.712
*Klebsiella pneumoniae*	0 (0.0)	5 (9.6)	0.318	0 (0.0)	5 (8.5)	1.000	4 (6.2)	1 (20.0)	0.318	4 (6.6)	1 (11.1)	0.620
*Raoultella ornithinolytica*	5 (27,8)	14 (26.9)	0.944	2 (18.2)	17 (28.8)	0.715	19 (29.2)	0 (0.0)	0.313	17 (27.9)	2 (22.2)	1.000
*Serratia odoferans*	1 (5.6)	8 (15.4)	0.430	0 (0.0)	9 (15.3)	0.336	7 (10.8)	2 (40.0)	0.120	8 (13.1)	1 (11.1)	1.000
*Staphylococcus aureus*	3 (16.7)	10 (19.2)	0.809	1 (9.1)	12 (20.3)	0.676	12 (18.5)	1 (20.0)	1.000	11 (18.0)	2 (22.2)	0.670

*Chi-square test was used to generate the p-values numbers in parenthesis are in %*.

*OR = 4.83 (95% CI: 1.15, 20.16).

*^+^OR = 6.1 (95% CI: 1.33, 28.57)*.

### Bacterial Isolates on the Ninth Day of Life and Associated Factors

**Figure 3:**
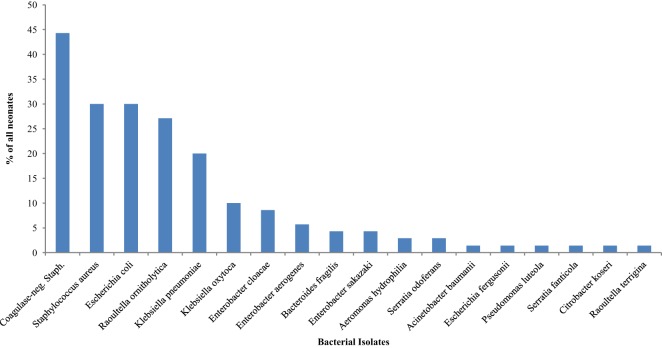
**Bacterial isolates from the rectum of neonates on the ninth day of life**.

The isolates obtained from all 70 (100.0%) neonates on the ninth day of life were as displayed in Figure 3. The most frequently isolated bacteria were coagulase-negative *Staphylococcus* (44.3%). Table 4 shows comparisons of bacterial isolates from the neonates by maturity, presence or absence of asphyxia at birth, mode of delivery, and whether or not exclusively breastfed for the first 9 days of life. Remarkably, the odds of isolating coagulase-negative *Staphylococcus* (OR = 4.17; 95% CI: 1.21, 7.38) was significantly higher in nonasphyxiated than asphyxiated neonates as shown in Table 4.

**Table 4:** **Frequency of 10 common bacterial isolates from the rectum of neonates on the ninth day of life**.

Isolates	Maturity			Asphyxia			Mode of delivery			Exclusive Breastfeeding		
	Preterm *N* = 18	Term *N* = 52	*p*	Yes *N* = 11	No *N* = 59	*p*	Vaginal *N* = 65	CS *N* = 5	*p*	Yes *N* = 61	No *N* = 9	*p*
*Bacteroides fragilis*	0 (0.0)	3 (5.8)	0.564	0 (0.0)	3 (5.1)	1.000	3 (4.6)	0 (0.0)	1.000	3 (4.9)	0 (0.0)	1.000
Coagulase-neg. *Staph*	7 (38.9)	24 (46.2)	0.784	8 (72.7)	23 (39.0)	0.039*	30 (46.2)	1 (20.0)	0.374	26 (42.6)	5 (55.6)	0.496
*Enterobacter cloacae*	3 (16.7)	3 (5.8)	0.172	0 (0.0)	6 (10.2)	0.580	5 (7.7)	1 (20.0)	0.370	6 (9.8)	0 (0.0)	1.000
*Enterobacter aerogenes*	0 (0.0)	4 (7.7)	0.586	0 (0.0)	4 (6.8)	1.000	3 (4.6)	1 (20.0)	0.262	4 (6.6)	0 (0.0)	1.000
*Enterobacter sakazakii*	1 (5.6)	2 (3.8)	0.758	2 (18.2)	1 (1.7)	0.062	3 (4.6)	0 (0.0)	1.000	3 (4.9)	0 (0.0)	1.000
*Escherichia coli*	6 (33.3)	15 (28.8)	0.770	5 (45.5)	16 (27.1)	0.223	18 (27.7)	3 (60.0)	0.155	17 (27.9)	4 (44.4)	0.437
*Klebsiella oxytoca*	1 (5.6)	6 (11.5)	0.668	1 (9.1)	6 (10.6)	1.000	7 (10.8)	0 (0.0)	1.000	6 (9.8)	1 (11.1)	0.905
*Klebsiella pneumoniae*	3 (16.7)	11 (21.2)	1.000	2 (18.2)	12 (20.3)	1.000	13 (20.0)	1 (20.0)	1.000	11 (18.3)	3 (33.3)	0.370
*Raoultella ornithinolytica*	5 (27.8)	14 (26.9)	1.000	1 (9.1)	19 (32.2)	0.159	19 (29.2)	0 (0.0)	0.313	17 (27.9)	2 (22.2)	1.000
*Staphylococcus aureus*	7 (38.9)	14 (26.9)	0.380	20 (29.0)	1 (100.0)	0.124	18 (27.7)	3 (60.0)	0.155	17 (27.9)	4 (44.4)	0.437

*Chi-square test was used to generate the p-values. Numbers in parenthesis are in %*.

*OR = 4.17 (95% CI: 1.21, 7.38).

### Bacterial Isolates on the 14th Day of Life and Associated Factors

**Figure 4:**
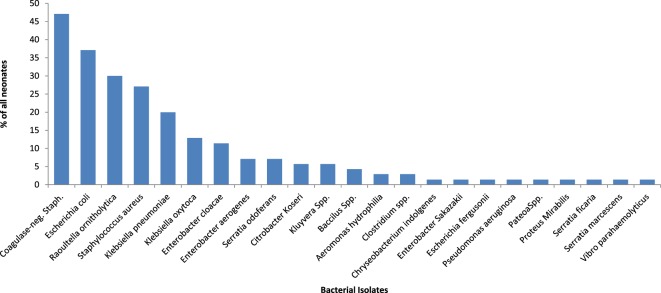
**Bacterial isolates from the rectum of neonates on the 14th day of life**.

Figure 4 and Table 5 show the bacteria isolates obtained from the samples of all 70 (100.0%) neonates on day 14. Coagulase-negative *Staphylococcus* (47.1%) remains the most frequently isolated organism, it was isolated more from those who were not exclusively breastfed than those who had exclusively breastfeeding indicating significantly higher odds of *Staphylococcus aureus* (OR = 7.38; 95% CI: 1.62, 33.61) from nonexclusively breastfed than that of exclusively breastfed neonates.

**Table 5:** **Frequency of 10 common bacterial isolates from the rectum of neonates on the 14th day of life**.

Isolates	Maturity			Asphyxia			Mode of delivery			Exclusive Breastfeeding		
	Preterm *N* = 18	Term *N* = 52	*p*	Yes *N* = 11	No *N* = 59	*p*	Vaginal *N* = 65	CS *N* = 5	*p*	Yes *N* = 61	No *N* = 9	*p*
Coagulase-neg. *Staph*	10 (55.6)	23 (44.2)	0.407	4 (36.4)	29 (49.2)	0.435	32 (49.2)	1 (20.0)	0.207	31 (50.8)	2 (22.2)	0.157
*Escherichia coli*	5 (27.8)	21 (40.4)	0.304	4 (36.4)	22 (37.3)	1.000	25 (38.5)	1 (20.0)	0.644	24 (39.3)	2 (22.2)	0.468
*Raoultella ornithinolytica*	6 (33.3)	9 (17.3)	0.153	3 (27.3)	12 (20.3)	0.691	13 (20.0)	2 (40.0)	0.290	12 (19.7)	3 (33.3)	0.392
*Staphylococcus aureus*	7 (38.9)	12 (23.1)	0.194	2 (18.2)	17 (28.8)	0.715	16 (24.6)	3 (60.0)	0.119	13 (21.3)	6 (66.7)	0.004*
*Klebsiella pneumoniae*	2 (11.1)	12 (23.1)	0.494	2 (18.2)	12 (20.3)	1.000	12 (18.5)	2 (40.0)	0.260	12 (19.7)	2 (22.2)	1.000
*Klebsiella oxytoca*	3 (16.7)	6 (11.5)	0.685	1 (9.1)	8 (13.6)	1.000	9 (13.8)	0 (0.00)	1.000	9 (14.8)	0 (0.0)	0.592
*Enterobacter cloacae*	2 (11.1)	6 (11.5)	1.000	2 (18.2)	6 (10.2)	0.602	7 (10.8)	1 (20.0)	0.465	6 (9.8)	2 (22.2)	0.272
*Enterobacter aerogenes*	1 (5.6)	4 (7.7)	1.000	1 (9.1)	4 (6.8)	1.000	4 (6.2)	1 (20.0)	0.318	5 (8.2)	0 (0.0)	1.000
*Serratia odoferans*	2 (11.1)	3 (5.8)	0.597	0 (0.0)	5 (8.5)	1.000	5 (7.7)	0 (0.0)	1.000	5 (8.2)	0 (0.0)	1.000
*Citrobacter Koseri*	3 (16.7)	1 (1.9)	0.050	1 (9.1)	3 (5.1)	0.504	3 (4.6)	1 (20.0)	0.262	3 (4.9)	1 (11.1)	0.431

*Chi-square test was used to generate the p-values. Numbers in parenthesis are in %*.

*OR = 7.38 (95% CI: 1.62, 33.61).

### Trend of Acquisition of the Leading Bacteria in the First 2 Weeks of Life

Table 6 shows the trend and comparisons of the difference in colonization patterns between days 1, 3, 9, and 14. It appears that bacteria colonization in the neonates became established at slightly different times. On day 1, bacteria isolates were predominantly facultative anaerobes comprising coagulase-negative *Staphylococcus, Staphylococcus aureus, Escherichia coli*, and *Enterobacteria* other than *Escherichia coli* forming a large contingent (*Enterobacter cloacae, Enterobacter aerogenes, Raoultella ornithinolytica*, and *Klebsiella oxytoca*). On days 3 and 9, the bacteria isolates were heterogeneous but still dominated by coagulase-negative *Staphylococcus* and the *Enterobacteria, Escherichia coli*, and *Raoultella ornithinolytica*. On day 14, there was more diversity in the isolates with 23 different species but the dominance of coagulase-negative *Staphylococcus* and *Staphylococcus aureus* was maintained. Most of the remaining constituent bacteria were other members of the *Enterobacteriaceae* family. Statistically significant increasing trends were observed for *Staphylococcus aureus* and *Klebsiella pneumoniae*, respectively, as the neonates got older.

**Table 6:** **Comparisons of the common bacterial isolates from 70 neonates on days 1, 3, 9, and 14**.

Isolates	Day 1 *n* (%)	Day 3 *n* (%)	Day 9 *n* (%)	Day 14 *n* (%)	p*
Coagulase-negative *Staph*	27 (38.6)	41 (56.6)	31 (44.3)	33 (47.1)	0.011
*Staphylococcus aureus*	22 (31.4)	13 (18.6)	21 (30.0)	19 (27.1)	0.797
*Escherichia coli*	13 (18.6)	25 (35.7)	21 (30.0)	26 (37.1)	0.088
*Raoultella ornithinolytica*	11 (15.7)	11 (15.7)	19 (27.1)	15 (21.4)	0.171
*Enterobacter cloacae*	9 (12.9)	9 (12.9)	6 (8.6)	8 (11.4)	0.761
*Enterobacter aerogenes*	7 (10.0)	7 (10.0)	4 (5.7)	5 (7.1)	0.822
*Klebsiella oxytoca*	6 (8.6)	2 (2.9)	7 (10.0)	9 (12.9)	0.104
*Acinetobacter baumannii*	2 (2.9)	1 (1.4)	1 (1.4)	0 (0.0)	0.201
*Aeromonas hydrophila*	2 (2.9)	0 (0.0)	2 (2.9)	2 (2.9)	0.213
*Bacteroides fragilis*	2 (2.9)	7 (10.0)	3 (4.3)	0 (0.0)	0.074
*Clostridium* spp.	2 (2.9)	3 (4.3)	0 (0.0)	2 (2.9)	0.243
*Citrobacter koseri*	1 (1.4)	1 (1.4)	1 (1.4)	4 (5.7)	0.523
*Klebsiella pneumoniae*	0 (0.0)	5 (7.1)	14 (20.0)	14 (20.0)	<0.001
*Serratia odoferans*	0 (0.0)	6 (8.6)	6 (8.6)	5 (7.1)	0.183

*Generated using chi-square test for trend.

## Discussion

This study showed that almost all (92.9%) neonates had rectal colonization by various bacteria on the first day of life and all subsequently became colonized by day 3. The bacteria isolates from rectal swabs were dominated by the phyla *Firmicutes, Proteobacteria*, and *Bacteroidetes*. However, the diversity of the bacteria composition was limited to 18 genera comprising *Aeromonas, Acinetobacter, Bacillus, Bacteroides, Chryseobacteria, Citrobacter, Clostridium, Enterobacter, Escherichia, Klebsiella, Kluyvera, Pantoea, Proteus, Pseudomonas, Raoultella, Serratia, Staphylococcus, and Vibrio*. The clinically important species amongst these bacteria were *Bacteroides fragilis, Clostridium* spp., coagulase-negative *Staphylococcus, Enterobacter cloacae, Escherichia coli, Klebsiella oxytoca, Klebsiella pneumoniae, Raoultella ornithinolytica*, and *Staphylococcus aureus*. These bacteria have the propensity for translocation into the blood stream and the resultant septicemia.

That the neonates had bacteria isolates on the first day of life is consistent with the report by Rotimi and colleagues, in Lagos Nigeria, about 30 years earlier (14) and Pakistan (20, 21). Conversely, reports from Europe showed lower bacterial colonization rates on the first day of life (20–22). These reports revealed the marked differences in the timing of bacterial colonization of rectum of neonates between developed and developing countries probably a reflection of the level of hygiene of the delivery environment (21).

The pattern of bacterial colonization, as shown by bacterial isolation in the rectal swabs on days 1, 3, 9, and 14, falls into the expected pattern of aerobes-facultative groups. The anaerobes were the initial colonizers of the neonates’ rectum, followed by sequential establishment of the expected anaerobic bacteria as earlier reported by Walker et al. (23) and Fanaro et al. (24). However, the composition of the individual bacteria in the present study differ from those found in European neonates (21, 23, 24). Notably, the trend observed in the first 2 weeks of life was that coagulase-negative *Staphylococcus* and *Staphylococcus aureus* were acquired from the first day of life, peaking on day 3 and remained dominant throughout the first 2 weeks of life.

The *Enterobacteriaceae* maintained the expected trend of colonization after the first day of life, but at a moderate level and the expected change to an anaerobic pattern had not occurred by the end of the second week. The findings in the present study are consistent with studies in Sweden (22) and France (25) that consistently reported rising rates of colonization by *Staphylococcus aureus* and coagulase-negative *Staphylococcus* but at variance with earlier reports from Nigeria (14), United Kingdom (26), and Sweden (27), which reported high rates of colonization with *Escherichia coli, Enterococcus*, and *Streptococcus* and moderate rates with *Staphylococcus* spp. A possible explanation for this trend is the practice of short hospital stay for newly delivered mothers, rooming-in, and improved hygiene may all have reduced exposure of infants to typical fecal bacteria such as *Escherichia coli* in the hospital (21). With low *Escherichia coli* colonization, the traditional skin bacteria including *Staphylococcus aureus* and coagulase-negative *Staphylococcus* might have expanded and become dominant due to lack of competition from other commensals.

The high predominance of *Staphylococcus* spp. in the present study has some clinical implications. The neonatal rectal carriage of *Staphylococcus* spp. may be the possible source of the blood infection, which may explain the dominance of *Staphylococcus aureus* as the cause of neonatal septicemia in our hospitals (28–30). However, despite the high colonization rate by *Staphylococcus* spp. in this study, none of the neonates with this isolate had clinical features suggestive of septicemia. The present study found no statistically significant association between maternal factors such as prolonged rupture of membrane (PROM), HIV status, and bacterial colonization of the rectum of the neonates. Nonetheless, a gross examination of the spectrum of isolates from these mothers with PROM and HIV infection showed that the pattern of isolates were similar to those of other mothers.

The higher rate of *Escherichia coli* colonization among preterm than term neonates may be as a result of acquisition from hospital staff, visitors, and the mothers. In general, preterm neonates cared for in specialized units, hygienically controlled, and more likely to be on antibiotics usually show delayed colonization and a limited number and diversity of bacteria (31). However, the preterm neonates in this study were neither admitted nor given antibiotics, they were nursed in cots besides their mothers in the postnatal wards. Also, the fact that nonexclusively breastfed neonates had higher colonization with *Staphylococcus aureus* than those exclusively breastfed corroborates the finding by Harmsen et al. in the Netherlands (32). Another likely source of *Staphylococcus aureus* was the vaginal canal during the delivery period and the skin of caregivers.

An important, but unexpected, finding in this study was the absence of bacteria from the genus *Actinobacteria*, specifically *Bifidobacteria* spp. which is consistent with the finding by Ekwempu et al. (13) who found neither *Bifidobacteria* nor *Lactobacillus* spp. among a population of children in Northern Nigeria. More recent studies have also questioned the dominance of *Bifidobacteria*, with many reporting very low to negligible quantity (33–35), though an earlier study in the same environment (14), albeit more than 25 years ago, reported high rates of colonization by *Bifidobacteria*. One possible explanation for the absence of *Bifidobacteria* in our data is that suppression of *Bifidobacteria* could occur in some instances of predominant growth of gram-negative bacteria and *Staphylococcus* spp. in neonates (22, 33) or as suggested by Brandt et al. (36) and Leke et al. (37) that the level of *Bifidobacteria* was probably under the cultural detection threshold. Another notable negative finding was the absence of *Lactobacillus* spp. The extent to which *Lactobacilli* colonize the intestines of neonates is controversial. Most studies that utilized traditional biochemical methods similar to the present study also reported low *Lactobacillus* colonization rates in infants (21).

The findings in our study need to be viewed and interpreted with caution bearing in mind the limitations of the methods used for bacteria identification. The microbiological culture methods used for bacterial flora identification could only detect cultivable organisms. Recent studies on the intestinal microbiome have used nonculture techniques that allow the identification of a large number of bacteria that may not grow on standard culture media. This limits the ability to compare the results of the present study with other recent studies performed by analyzing 16S RNA or by metagenomics (38). Also, the fact that the population of neonates who participated in the study were recruited from a tertiary hospital limits the generalizability of the findings. The differences that may exist between neonates delivered elsewhere such as home, private, secondary, and primary health facilities compared with the sampled population remain largely undetermined.

## Conclusion

In all, using standard culture media, 12 species of bacterial flora were isolated from the stool of neonates delivered in Ibadan, Nigeria. These were *Bacillus, Bacteroides, Candida, Clostridium, Staphylococcus, Enterobacter, Enterobacteriaceae, Klebsiella, Proteus, Pseudomonas, Raoultella*, and *Serratia. Staphylococcus aureus* and *Escherichia coli* were the predominant isolates from the rectum of Nigerian neonates, and these isolates were influenced by breastfeeding and mild–moderate asphyxia. In all, bacterial diversity in the rectum increased as the neonates got older.

## Author Contributions

AO, AK, and OA conceptualised the study. OT, GO, and DO contributed to the study design. AO analyzed the data and wrote the first draft of the manuscript. All the authors critically review and contributed to the final draft of the manuscript.

## Conflict of Interest Statement

The authors declare that the research was conducted in the absence of any commercial or financial relationships that could be construed as a potential conflict of interest.
